# Supplementary material for: Benthic communities at two remote Pacific coral reefs: effects of reef habitat, depth, and wave energy gradients on spatial patterns
Source: PeerJ. 2013 May 28;1:e81. doi: 10.7717/peerj.81 (PMC3669270; doi:10.7717/peerj.81)
Supplement: Supplemental Information — Benthic percent cover values identified to the lowest taxonomic resolution possible across reef habitats and depths at Kingman Reef and Palmyra Atoll and subsequent allocation success values for formal testing of group differences. [file peerj-01-81-s001.docx]

**Supplementary Table 1.** Summary of functional group benthic community patterns and variations in individual taxa at Kingman Reef and Palmyra Atoll across reef habitats (patch reef, backreef, forereef, reef terrace) and depths (5, 10, 20 m). Mean +1 standard error (se) percentage cover values are shown. A blank cell indicates 0.0% cover. *Pachy/Stereo* = *Pachyclavularia*/*Stereonephthya*.

**Supplementary Table 2.** Results of CAP analyses examining within-island variations in benthic communities at three taxonomic resolutions at Kingman Reef and Palmyra Atoll. m, the number of principal coordinate (PCO) axes used in the CAP procedure; %Var, percentage of the total variance explained by the first *m* PCO axes; allocation success, the percentage of points correctly allocated into each group; δ^2^, squared canonical correlation.
